# First-Line Treatment Strategies in IMDC Favourable-Risk Metastatic Clear Cell Renal Cell Carcinoma

**DOI:** 10.32604/or.2026.077711

**Published:** 2026-07-16

**Authors:** Alejandro Valdés, Jaime González-Montero, Carlos Rojas, Mauricio Burotto

**Affiliations:** 1 Bradford Hill Clinical Research Center, Santiago, Chile; 2 Instituto Nacional del Cáncer, Santiago, Chile; 3 Faculty of Medicine, Finis Terrae University, Santiago, Chile

**Keywords:** Renal cell carcinoma, clear cell renal carcinoma, favourable risk, IMDC, TKI, VEGF-TKI, immunotherapy, IO, first-line treatment, active surveillance

## Abstract

Immune checkpoint inhibitors (ICIs) combined with vascular endothelial growth factor tyrosine kinase inhibitors (VEGF-TKIs) have transformed the treatment landscape of advanced clear cell renal cell carcinoma (ccRCC). Current guidelines favour ICI plus VEGF-TKI (IO+TKI) combinations for favourable-risk disease (International Metastatic RCC Database Consortium [IMDC] score 0) based on improved objective response rates and progression-free survival. However, no IO+TKI combination has demonstrated a statistically significant overall survival (OS) benefit in this subgroup. A pooled analysis of four pivotal phase III trials (n = 839 favourable-risk patients) revealed no OS advantage for IO+TKI versus sunitinib monotherapy (hazard ratio [HR] 1.24; 95% CI 0.86–1.78) despite higher toxicity rates (71–82% Grade ≥ 3 adverse events vs. 63–72% with sunitinib) and substantially greater cost. The IMDC favourable-risk category represents approximately 20% of metastatic ccRCC cases and is often characterised by indolent disease biology. Emerging molecular classifications reveal distinct transcriptomic subgroups, including an angiogenic subtype (ccA/CC-e.2/clusters 1–2) enriched in favourable-risk patients, characterised by high hypoxia-inducible factor (HIF) pathway gene expression, frequent PBRM1 mutations, robust VEGF-TKI responsiveness, and comparatively lower benefit from immunotherapy. Current clinical risk stratification fails to capture this molecular heterogeneity, limiting optimal treatment selection. VEGF-TKI monotherapy (median OS 47.6–79.4 months) and active surveillance remain valid, evidence-based alternatives in carefully selected favourable-risk patients, particularly those with asymptomatic, metachronous, or otherwise indolent disease. Uncritical universal use of IO+TKI in this population may therefore represent overtreatment. The development and validation of predictive biomarkers, refinement of molecular risk stratification, and exploration of novel agents with more favourable toxicity profiles (e.g., HIF-2α inhibitors) are urgently required to personalise therapy and identify candidates for rational treatment de-escalation.

## 1 Introduction

Renal cancer accounts for approximately 2% of all malignancies, with a global estimate of 434,419 new cases (14th) and 155,702 deaths (16th) annually [1,2]. Renal cell carcinoma (RCC) comprises > 90% of kidney cancers, with clear cell RCC (ccRCC) as the most common subtype (70%). The remaining subtypes are grouped as non–clear cell RCCs, of which papillary and chromophobe carcinomas are the most frequent [3].

Localised disease is managed with partial or radical nephrectomy; however, approximately 30% of patients present with metastases at diagnosis, and an additional 30% experience recurrence following nephrectomy [4]. Prognosis in advanced disease remains poor, with a five-year survival rate near 15% [5]. Historically considered chemoresistant, RCC treatment saw its first meaningful gains with cytokine therapy (interferon-α and interleukin-2), which produced low overall response rates (ORRs) and substantial toxicity, although a minority of patients experienced durable benefits [6]. The advent of vascular endothelial growth factor receptor (VEGF) tyrosine kinase inhibitors (VEGF-TKIs) transformed care. Immunotherapy with immune checkpoint inhibitors (ICIs) targeting PD-1/PD-L1 and CTLA-4 further improved outcomes.

First-line therapy selection in advanced ccRCC is often guided by the International Metastatic RCC Database Consortium (IMDC) criteria—developed during the advent of VEGF-TKI therapies—which stratify patients into favourable, intermediate, and poor risk groups [7]. Approximately 20% of patients are favourable-risk, typically with a more indolent disease presentation [8]. Current guidelines recommend combination regimens: ICI with VEGF-TKI (IO+TKI) or dual ICI (anti–PD-1 with anti–CTLA-4; IO+IO) [9,10,11]. In intermediate–poor-risk disease, these combinations confer a median overall survival (mOS) advantage over TKI monotherapy. Guidelines recommend IO+TKI in favourable-risk patients due to higher ORR and longer median progression-free survival (mPFS) versus TKI alone; however, the absence of a consistent mOS advantage, in addition to increased toxicity and cost, argues against a one-size-fits-all approach. In carefully selected scenarios, TKI monotherapy, IO+IO combinations, or active surveillance (AS) remain reasonable options. Emerging molecular classifications suggest biologically distinct subgroups with differing angiogenic and immune signatures, with potential implications for therapy selection [12]. The development and validation of predictive biomarkers are therefore urgently required to refine treatment selection and personalise care.

## 2 Characterisation of Favourable-Risk ccRCC

The current definition of risk groups relies on prognostic models developed in the pre-immunotherapy era. Numerous studies have identified prognostic factors in renal cancer to refine outcome estimation and help trial stratification. The most widely used tools—the Memorial Sloan Kettering Cancer Center (MSKCC) model [13,14] (developed during the cytokine therapy era) and the IMDC criteria [8]—utilise clinical and laboratory variables to estimate prognosis (Table 1). Both tools categorise patients into three risk groups (favourable, intermediate, and poor) based on pre-treatment prognostic features. The IMDC model has been externally validated in diverse populations [15,16]. Further refinement of the IMDC database identified a ‘very favourable’ subgroup [17]. The following variables were independently associated with OS in a multivariable analysis (hazard ratio [HR] approximately 1.4–1.5; *p* < 0.05): time from initial diagnosis to systemic therapy (<3 vs. ≥3 years), Karnofsky Performance Status (80 vs. >80%), and the presence of brain, liver, or bone metastases. Patients were classified as very favourable (0 risk factors; 29%) or favourable (≥1 risk factor; 71%), with an mOS of 64.8 vs. 45.6 months, respectively (HR 1.84; *p* < 0.001). This new group underwent external validation in a Turkish cohort [18].

Developed during the VEGF-TKI era, the IMDC criteria demonstrated the advantages of VEGF-TKI therapies across all risk groups, albeit with poorer outcomes in the intermediate and poor categories. Contemporary ccRCC trials evaluating immunotherapy combinations (IO+IO or IO+TKI), therefore continue to stratify patients by IMDC risk. Retrospective analyses from the IMDC database suggest that the model retains prognostic discrimination in patients treated with IO-based doublets, supporting its ongoing use as a robust prognostic tool [19].

In practice, IMDC risk is often—imperfectly—treated as a predictive biomarker to guide first-line regimen selection [20]. However, IMDC was designed to estimate disease aggressiveness and overall survival primarily independently of the therapy received, not to predict differential benefit between treatments. Its use as a predictive biomarker is based partially on the initial results of CheckMate 214 [21], in which sunitinib outperformed nivolumab plus ipilimumab in the favourable-risk subgroup. Establishing a predictive biomarker of treatment benefit requires a formal test of interaction between biomarker status (positive vs. negative) and treatment effect across at least two therapies (or therapy vs. placebo) [22]. IO+TKI doublets have not improved mOS over TKI monotherapy in favourable-risk disease [23], and updated CheckMate 214 data show a nonsignificant trend towards improved mOS with IO+IO versus TKI [21]. Moreover, a retrospective analysis of CheckMate 214 suggests that the prognostic performance of IMDC attenuates over time and with IO exposure, raising questions about its long-term prognostic capability in the immunotherapy era [24]. Extrapolating IMDC categories as a predictive biomarker, therefore, risks conflating prognosis with genuine treatment-effect modification.

Whether newer models should remain purely prognostic or evolve to incorporate predictive elements remains an open question. Some IMDC variables (e.g., anaemia, thrombocytosis, neutrophilia) are surrogates of systemic inflammation and may correlate with tumour biology; clinical variables alone cannot capture underlying molecular drivers. Genomic profiling may enhance risk discrimination and is an active area of investigation. For example, retrospective analyses of COMPARZ and RECORD-3 identified mutations in *BAP1*, *PBRM1*, and *TP53* as independent prognostic factors that improved stratification in addition to the MSKCC criteria [25]. Nonetheless, integrating molecular predictive biomarkers into routine care presents practical obstacles, including cost, assay availability, analytical validity, and uncertain incremental clinical utility [26].

**Table 1:** The Memorial Sloan Kettering Cancer Center (MSKCC) and International Metastatic RCC Database Consortium (IMDC) prognostic models.

A	MSKCC Model (also Known as the Motzer Criteria)	IMDC Model (also Known as the Heng Criteria)
Patient cohort and data derivation	Single-centre retrospective analysis. 670 patients treated between 1975 and 1996; majority received interferon or interleukin. Other patients received ineffective therapies (chemotherapy or hormonal therapy).	Multicentre international database (IMDC). 645 patients with metastatic RCC treated with vascular endothelial growth factor (VEGF)–targeted agents (sunitinib, sorafenib, or bevacizumab plus interferon) between August 2004 and July 2008.
Risk factors	Low Karnofsky Performance Status (KPS < 80%) Anaemia (<LLN) High lactate dehydrogenase (LDH > 1.5 × ULN) Hypercalcaemia (corrected calcium > ULN) Short interval from diagnosis to systemic treatment (<1 year)	Low Karnofsky Performance Status (KPS < 80%) Anaemia (<LLN) Hypercalcaemia (corrected calcium > ULN) Neutrophilia (elevated neutrophil count) (>ULN) Thrombocytosis (>ULN) Short interval from diagnosis to systemic treatment (<1 year)
Risk group classification	Favourable: 0 factors Intermediate: 1–2 factors Poor: ≥3 factors	Favourable: 0 factors Intermediate: 1–2 factors Poor: ≥3 factors
Prognostic significance (median OS)	Favourable risk: 29.6 months (95% CI 20.9–37.8 months); 3-year survival 45% Intermediate risk: 13.8 months (95% CI 12.4–15.9); 3-year survival 17% Poor risk: 4.9 months (95% CI 4.3–6.3); 3-year survival 2%	Favourable risk: 43.2 months (95% CI 31.4–50.1) Intermediate risk: 22.5 months (95% CI 18.7–25.1) Poor risk: 7.8 months (95% CI 6.5–9.7)

## 3 Refining the Definition of Favourable Risk

### 3.1 Biology of ccRCC

Most ccRCCs are initiated by biallelic *VHL* inactivation, typically through loss of chromosome 3p followed by somatic mutation or promoter methylation [27]. *VHL* encodes pVHL, the substrate-recognition subunit of an E3 ubiquitin ligase complex which targets prolyl-hydroxylated HIF-α for proteasomal degradation [28]. The loss of pVHL stabilises HIF-α—predominantly HIF-2α in ccRCC—which dimerises with ARNT (HIF-1β) and drives a pseudohypoxic program, upregulating *VEGF*, *EPO*, *CA9*, and *GLUT1* and promoting angiogenesis and metabolic reprogramming [29,30].

The loss of pVHL alone is usually insufficient for malignant transformation; co-alterations in other 3p tumour suppressors modulate phenotype and prognosis. *PBRM1*, *SETD2*, and *BAP1* encode epigenetic regulators which define partially distinct molecular and clinicopathologic subsets [31]. *PBRM1* (mutated in 41–50% of cases) encodes BAF180, a PBAF SWI/SNF complex subunit involved in chromatin remodelling; its loss is common in more indolent tumours and is associated with relatively favourable outcomes [32]. *SETD2* and *BAP1* (mutated in 13–28% and 11–17% of cases, respectively), which regulate H3K36me3 and H2AK119ub1, respectively, are enriched in higher-grade, more aggressive disease and portend inferior survival [33,34]. *KDM5C*, an X-linked H3K4 demethylase which mutates more often in male tumours, is implicated in metabolic reprogramming and increased tumorigenicity, aligning with adverse biology in some series [35].

An inflamed tumour microenvironment with T-cell infiltration and variable PD-L1 expression are often observed in ccRCC; myeloid-driven immunosuppression and T-cell dysfunction are also common, supporting initial sensitivity and adaptive resistance to immune checkpoint blockade [36,37]. Angiogenic, T-effector, and myeloid-inflammatory signatures have shown distinct associations with response to VEGF-targeted therapy or immunotherapy-based combinations [38]. Marked intratumour heterogeneity with branched evolution is characteristic: truncal events such as 3p loss and *VHL* inactivation are shared across regions, whereas *PBRM1*, *SETD2*, *BAP1*, and *KDM5C* mutations and arm-level copy-number alterations are often subclonal [39,40,41].

### 3.2 Molecular Subgroups

The clinical heterogeneity of ccRCC has a clear molecular basis, with multi-omics studies defining reproducible subgroups differing in gene expression programs, dominant mutations, epigenetic features, and clinical behaviour [12,42]. The earliest widely adopted transcriptomic classification identified two main subtypes: clear cell A (ccA) and clear cell B (ccB) [43]. Overexpression of hypoxia-, angiogenesis-, and metabolism-related genes was observed in ccA tumours, which were associated with better survival than ccB. A subsequent meta-analysis of 480 tumours validated the ccA/ccB subtypes and identified a third VHL wild-type subgroup (Cluster 3) [44].

The Cancer Genome Atlas (TCGA) program integrated mRNA, miRNA, DNA methylation, copy-number, and proteomic data from 446 nephrectomy specimens and defined four mRNA (m1–m4) and four miRNA (mi1–mi4) clusters [45]. Three integrative subtypes (CC-e.1, CC-e.2, CC-e.3) were subsequently described across 894 RCCs. In ccRCC, CC-e.2 aligned with the angiogenic ccA subtype and had the best survival, CC-e.3 aligned with the more aggressive ccB subtype and had the worst outcomes, and CC-e.1 exhibited intermediate features. A French group subsequently linked transcriptomic subtypes to VEGF-targeted therapy in 53 primary tumours from patients later administered sunitinib [46]. Four clusters (ccrcc1–ccrcc4) were identified, alongside analyses of copy-number, methylation, and *VHL*/*PBRM1* mutations. The ccrcc2 subgroup showed an angiogenic/metabolic gene signature, overlapped with ccA, and had a good prognosis.

More recently, a multi-omics analysis of pre-treatment samples from the IMmotion151 trial (625 primary, 198 metastatic) defined seven RNA-based clusters [38]. Clusters 1 (angiogenic) and 2 (angiogenic–stromal) were enriched in MSKCC/IMDC favourable-risk patients, VEGF pathway activation, and *PBRM1*/*KDM5C* mutations. In IMmotion151 (atezolizumab–bevacizumab vs. sunitinib), these angiogenic clusters derived similar benefit from both treatment arms; a post-hoc analysis of JAVELIN Renal 101 (axitinib–avelumab versus sunitinib) showed improvement in mPFS with VEGF–IO doublets across all clusters [47].

Across transcriptomic classification systems, a reproducible angiogenic favourable subtype emerges, corresponding broadly to ccA, TCGA CC-e.2, CIT ccrcc2/3 and IMmotion clusters 1–2. This subtype is characterized by strong HIF–VEGF axis activity, relative paucity of immune infiltration, and frequent *PBRM1* mutations, and is associated clinically with favourable early-to-intermediate outcomes and substantial sensitivity to VEGF-targeted agents. Conversely, durable benefit from immune checkpoint blockade appears less common in this context. However, IMDC favourable-risk status is not synonymous with this angiogenic molecular phenotype, as a proportion of clinically favourable-risk tumours display immune-inflamed biology and may benefit more from IO-based combinations. Incorporating simple biomarkers, including angiogenesis gene signatures, *PBRM1* mutation status, and pragmatic surrogates of immune infiltration, could refine the definition of ‘favourable-risk’ and help identify patients suitable for de-escalation strategies such as AS and VEGF-TKI monotherapy.

### 3.3 The Search for Predictive Biomarkers

Currently, there is no validated predictive biomarker in clinical use for the selection of IO or TKI in ccRCC treatment [48]. Biomarker discovery remains a challenge because the most frequent genomic alterations involve tumour suppressor genes, and the predominance of loss-of-function events limits the identification of directly actionable predictive targets [49]. This challenge is compounded by pronounced spatial and temporal intratumour heterogeneity. Although alterations in genes such as *PBRM1*, *SETD2*, and *BAP1* may carry biologic and prognostic significance, their association with treatment benefit has been inconsistent across studies, and no single genomic alteration has entered clinical practice as a predictive biomarker [32].

Traditional markers of immunotherapy response have no role in ccRCC; tumour mutation burden is usually low, and PD-L1 is not useful in clinical practice. There is a discrepancy in PD-L1 positivity among clinical trials [50]. Sarcomatoid differentiation repeatedly demonstrates a disproportionate response to IO-containing regimens compared with sunitinib; however, formal, prespecified interaction testing to establish it as a predictive biomarker is lacking [51]. Kidney injury molecule-1 (KIM-1), a transmembrane glycoprotein overexpressed in ccRCC, is a promising biomarker. A phase III trial (IMmotion010) reported that patients with high serum KIM-1 had worse disease-free survival (DFS); however, DFS improved following treatment with adjuvant atezolizumab versus placebo (HR 0.72, 95% CI 0.52–0.99) [52]. A post-hoc analysis of the CheckMate 214 trial showed similar results, with worse DFS both in the IO and TKI arms [53]. A reduction in serum KIM-1 after a single cycle of nivolumab plus ipilimumab was associated with long-term efficacy of this IO doublet.

Multiple biomarker modalities are under active study in ccRCC, including liquid biopsy with circulating tumour DNA (ctDNA) and circulating tumour cells (CTCs), microbiome profiling, single-cell/spatial transcriptomics, and radiomics. Each offers complementary windows to tumour biology and the tumour–host interface [48,54]. Machine-learning radiomics applied to routine baseline CT imaging appears feasible for discriminating responders vs. non-responders to PD-1 blockade, providing a scalable “digital biomarker” that can be paired with clinical features for individualised risk–benefit decisions [55]. In parallel, liquid biopsy dynamics—particularly early on-treatment changes in circulating tumour DNA—have demonstrated potential as a real-time, minimally invasive marker of ICI response, enabling earlier identification of primary resistance and adaptive sequencing strategies [56].

Transcriptomic programs related to angiogenesis, T-effector activity, and myeloid inflammation have improved understanding of the biologic determinants of response in ccRCC, although their predictive value has been inconsistent across IO+IO and IO+TKI studies [57,58,59]. Collectively, these observations suggest that future biomarker development in ccRCC will likely depend on integrated models rather than any single assay. In this context, incorporation of accessible molecular and microenvironmental features, including angiogenesis-related gene signatures, *PBRM1* status, and pragmatic surrogates of immune infiltration, may help refine the biologic definition of favourable-risk disease and better identify patients most suitable for de-escalation strategies such as active surveillance or VEGF-targeted monotherapy.

## 4 Systemic Treatment for Favourable-Risk Advanced Disease

### 4.1 TKI Monotherapy

VEGF-TKIs transformed the therapy of metastatic ccRCC after the interleukin era. Phase III trials of sunitinib [60,61], pazopanib [62,63], and sorafenib [64,65] demonstrated improvements in mPFS and ORR, but only sunitinib improved mOS. The IMDC model was developed in a cohort of 645 patients with metastatic RCC administered VEGF-targeted therapy (sunitinib, sorafenib, or bevacizumab plus interferon), reporting an mOS of 29.6 months (95% CI, 20.9–37.8) and a 3-year survival of 45% in favourable-risk patients [8]. Across phase III trials using sunitinib as the control group, mPFS ranged from 12.9 to 20.6 months and mOS from 47.6 to 79.4 months within the favourable-risk population (Table 2). Sunitinib and pazopanib remain in clinical use; sorafenib has largely been abandoned due to inferior efficacy and an unfavourable toxicity profile. Next-generation VEGF-TKIs have also been investigated in the first-line setting. A phase III trial of axitinib [66] and the phase III TIVO-1 trial [67] with tivozanib failed to improve mOS compared with sorafenib.

COMPARZ [68], a noninferiority randomised phase III trial, compared first-line sunitinib with pazopanib in advanced ccRCC. Pazopanib achieved an mPFS of 8.4 months versus 9.5 months with sunitinib (HR, 1.05; 95% CI, 0.90–1.22), meeting the noninferiority margin (upper bound of the 95% CI < 1.25); mOS was 28.4 versus 29.3 months (HR, 0.91; 95% CI, 0.76–1.08) and ORR was similar (33% with pazopanib [1 CR] vs. 29% with sunitinib [3 CR]). Toxicity profiles differed: sunitinib was associated with more fatigue, hand–foot syndrome, mucositis, and haematologic adverse events; pazopanib caused more hepatotoxicity and weight loss. Quality-of-life (QoL) scores favoured pazopanib in 11 of 14 health domains. In the PISCES crossover preference study [69], 70% of patients preferred pazopanib (citing less fatigue and better overall QoL), 22% preferred sunitinib (frequently citing less diarrhoea), and 8% had no preference (*p* < 0.001). Real-world data show similar mOS between first-line sunitinib vs. pazopanib when adjusted for IMDC criteria [70].

Multiple sunitinib dosing schedules have been explored [71,72]. The pivotal phase III trial recommended 50 mg once daily for 4 weeks on and 2 weeks off (4/2). Continuous daily dosing did not improve the efficacy of the 4/2 schedule [73]. Phase II studies of an alternative 2/1 regimen demonstrated similar efficacy with reduced toxicity [74,75,76]. Treatment breaks (‘stop-and-go’ strategy) trials have sought to improve QoL and reduce cost. The open-label, noninferiority phase II/III STAR trial [77] randomised 920 patients to conventional continuous therapy with pazopanib or sunitinib or a drug-free-interval strategy after 24 weeks of treatment until radiographic progression [78]. Noninferiority for OS was attained in the intention-to-treat analysis (HR, 0.94; 95% CI, 0.80–1.09) but not within the prespecified margin in the per-protocol population. Noninferiority in quality-adjusted life-years was achieved in both the intention-to-treat and per-protocol analyses (per-protocol marginal difference, 0.04).

### 4.2 Immunotherapy (IO+IO)

The phase III CheckMate 214 trial compared four cycles of nivolumab plus ipilimumab, followed by nivolumab alone, versus sunitinib [79]. Randomisation was stratified by IMDC risk. Coprimary endpoints (ORR, mPFS, and mOS) were assessed in the intermediate-/poor-risk cohort; analyses in favourable-risk patients were prespecified as exploratory. In intermediate-/poor-risk disease, nivolumab plus ipilimumab significantly improved ORR (including complete responses [CR]) and mOS. Although mPFS was numerically longer, the difference did not reach statistical significance. These benefits were maintained over extended follow-up [21]. Sunitinib demonstrated higher ORRs and mPFS in the favourable-risk subgroup (Table 2), resulting in guideline restriction of nivolumab plus ipilimumab to IMDC intermediate/poor-risk patients. Notably, the initial advantage of sunitinib attenuated with longer follow-up: a greater proportion of patients achieved CR with nivolumab plus ipilimumab (12.8% vs. 6.5%), with mOS trending longer (77.9 vs. 66.7 months; HR 0.82, 95% CI 0.60–1.13).

In the phase II PRISM trial [80], reduced-frequency ipilimumab plus nivolumab lowered grade 3–5 toxicity versus standard scheduling (32.8% vs. 53.1%; OR 0.43), but failed to meet the prespecified 12-month PFS efficacy threshold. Whether first-line nivolumab requires concurrent anti–CTLA-4 remains uncertain. Adaptive phase II trials—OMNIVORE [81], HCRN GU16-260 [82], and TITAN-RCC [83]—tested deferred salvage ipilimumab after nivolumab, but cross-trial interpretation is limited by population and design heterogeneity. A pooled analysis found deferred ipilimumab had limited efficacy and feasibility, supporting upfront nivolumab plus ipilimumab as standard therapy [84].

### 4.3 TKI+IO

Following the efficacy of dual checkpoint blockade in CheckMate 214, multiple phase III randomised trials compared IO+TKI combinations with sunitinib monotherapy. The addition of an ICI to a VEGF-directed TKI aims to extend durable disease control in a subset of patients; distinct mechanisms of action may delay resistance, and preclinical data support potential synergy between anti-angiogenic therapy and immunotherapy [85,86].

Across the ccRCC population, the following three regimens (vs. sunitinib) consistently improved ORR, mPFS, and mOS: pembrolizumab + axitinib in KEYNOTE-426 [87,88,89,90], nivolumab + cabozantinib in CheckMate-9ER [91,92,93,94], and lenvatinib + pembrolizumab in CLEAR [95,96,97] (Table 2). In JAVELIN Renal 101, avelumab + axitinib increased ORR and mPFS, but without a statistically significant mOS difference [98,99,100,101,102].

Within the IMDC favourable-risk subgroup, the main benefits are confined to higher ORR and longer mPFS. Across trials, a consistent OS advantage over sunitinib has not been demonstrated, with hazard ratios commonly near unity. These subgroup findings should be interpreted cautiously and regarded as hypothesis-generating rather than definitive. The lack of OS benefit is partly explained by the long natural history of favourable-risk disease and the low number of deaths, making OS analyses underpowered. In addition, the control arm is comparatively strong because favourable-risk tumours are often angiogenesis-high and VEGF-sensitive, consistent with the association between an angiogenesis signature and outcomes on sunitinib in KEYNOTE-426 (Rini et al. 2025). OS separation may be further attenuated by effective post-progression therapy—typically an ICI—and by the indolent biology of many tumours, which reduces the likelihood that early intensification translates into an OS gain [89]. Supporting this, an exploratory pooled analysis of favourable-risk patients (n = 839) from KEYNOTE-426, CheckMate-9ER, CLEAR, and JAVELIN Renal 101 reported no OS benefit for IO+TKI versus sunitinib (HR 1.24; 95% CI 0.86–1.78) [103]. Meta-analyses are concordant, finding no significant OS improvement in favourable-risk disease and highlighting the need for longer follow-up and more mature data [23,104]. Real-world evidence from ARON-1, a retrospective cohort of 524 favourable-risk patients, likewise showed no difference in median OS between sunitinib and IO+TKI combinations (*p* = 0.761) [105].

**Table 2:** Results from phase III IO+IO and IO+TKI trials, including favourable-risk patients.

Trial	CheckMate 214 [21,79]	CheckMate 9ER [91,92,93,94]	KEYNOTE-426 [87,88,89,90]	CLEAR (Study 307) [95,96,97]	JAVELIN Renal 101 [98,99,100,101,102]
Intervention vs. comparator	Nivolumab 3 mg/kg + ipilimumab 1 mg/kg every 3 weeks for 4 doses, followed by nivolumab 3 mg/kg every 2 weeks vs. sunitinib 50 mg daily for 4 weeks (6-week cycle).	Nivolumab + cabozantinib vs. sunitinib 50 mg daily for 4 weeks (6-week cycle).	Pembrolizumab 200 mg q3w + axitinib 5 mg bid vs. sunitinib 50 mg daily for 4 weeks (6-week cycle).	Lenvatinib 20 mg qd + pembrolizumab 200 mg q3w (L+P) vs. lenvatinib 18 mg qd + everolimus 5 mg qd (L+E) vs. sunitinib 50 mg daily for 4 weeks (6-week cycle) (S) (1:1:1).	Avelumab 10 mg/kg + axitinib 5 mg bid vs. sunitinib 50 mg daily for 4 weeks (6-week cycle).
Total number of patients (n, %)	1096 (249, 23%)	651 (146, 22%)	861 (269, 31.2%)	1069 (348, 32%)	886 (196, 22%)
ORR overall population (%)	39.5 vs. 33.0 (12.0 vs. 9.0).	56 vs. 28 (13.3 vs. 4.9).	60.5 vs. 39.6 (11.6 vs. 4.0).	L+P vs. S: 71.3 vs. 36.7 (18.3 vs. 4.8).	64.8 vs. 31.4 (7.8 vs. 4.5).
ORR favourable risk (CR) %	29.6 vs. 51.6 (12.8 vs. 6.5).	67.6 vs. 45.8 (16.2 vs. 9.7).	68.8 vs. 50.4 (13 vs. 6.1).	L+P vs. S: 68.2 vs. 50.8 (20.9 vs. 4.8).	75.5 vs. 48.8 (9.6 vs. 5.2).
mPFS overall population, months (HR, 95%CI)	12.4 vs. 12.3 (0.88, 0.75–1.03).	16.6 vs. 8.4 (0.59, 0.49–0.71).	15.7 vs. 11.1 (0.69, 0.59–0.81).	L+P vs. S: 23.9 vs. 9.2 (0.47, 0.38–0.57; *p* < 0.0001).	13.9 vs. 8.5 (0.66, 0.57–0.77; *p* < 0.0001).
mPFS favourable risk, months (HR, 95%CI)	12.8 vs. 20.6 (1.03, 0.77–1.37).	21.4 vs. 13.9 (0.76, 0.57–1.02)	20.7 vs. 17.9 (0.76, 0.57–1.02).	L+P vs. S: 28.6 vs. 12.9 (0.50, 0.35–0.71).	20.7 vs. 13.8 (0.75, 0.53–1.07; *p* = 0.1109).
OS overall population, months (HR, 95%CI)	52.7 vs. 37.8 (0.72, 0.62–0.83).	49.5 vs. 35.5 (0.70, 0.56–0.87).	47.2 vs. 40.8 (0.84, 0.71–0.99).	L+P vs. S: 53.7 vs. 54.3 (0.79, 0.63–0.99; *p* = 0.0424).	44.8 vs. 38.9 (0.88, 0.75–1.04; *p* = 0.0669).
OS favourable risk, months (HR, 95%CI)	77.9 vs. 66.7 (0.82, 0.60–1.13).	NR vs. 47.6 (1.07, 0.63–1.79).	60.3 vs. 62.4 (1.10, 0.79–1.54).	L+P vs. S: NR vs. 59.9 (0.94, 0.58–1.52).	79.4 vs. 65.5 (0.73, 0.48–1.10; *p* = 0.129).
Adverse events (%)	All grades 93.0 vs. 97.0 Grade ≥ 3 46.0 vs. 63.0	All grades 99.7 vs. 99.1 Grade ≥ 3 75.3 vs. 70.6	All grades 98.4 vs. 99.5 Grade ≥ 3 75.8 vs. 70.6	All grades L+P vs. S: 99.7 vs. 98.5 Grade ≥ 3 L+P vs. S: 82.4 vs. 71.8	All grades 99.5 vs. 99.3 Grade ≥ 3 71.2 vs. 71.5
QoL	Improved	Similar	Similar	Similar	Not reported

Note: the CLEAR trial was stratified by Memorial Sloan Kettering Cancer Center risk group; IMDC-stratified results are included.

### 4.4 Active Surveillance

Certain patients exhibit indolent tumour growth kinetics and may be suitable for AS in lieu of immediate systemic therapy, deferring treatment-related toxicity. Economic benefit is uncertain, as many patients ultimately require therapy. Potential risks include anxiety or reduced QoL in some individuals who prefer active intervention, and the possibility of clinical compromise if rapid tumour acceleration occurs. Prospective and retrospective studies have established AS as a safe and valid alternative [106,107].

A single-arm phase II trial was conducted on asymptomatic, treatment-naïve metastatic RCC (including non–clear cell histologies; prior surgery or radiotherapy allowed) [108]. Management consisted of observation with treatment initiation at the joint discretion of the clinician and patient; progression alone did not mandate stopping AS. Of the 48 enrolled patients (96% clear cell; 98% prior nephrectomy; 77% IMDC intermediate/poor risk), the median AS duration was 14.9 months (95% CI 10.6–25.0) with 38 months median follow-up. Overall, 90% experienced progression (median time to progression 9.4 months; 95% CI 7.4–13.4), and mOS was 44.5 months (95% CI 37.6–NR). Multivariable analyses delineated two prognostic strata: a favourable group (60%) with 0–1 IMDC factors and metastases confined to 1–2 organs (median AS 22.2 months; 95% CI 13.8–33.3) and an unfavourable group (all others; median AS 8.4 months; 95% CI 3.2–14.1; *p* = 0.0056). Patient-reported anxiety/depression and FKSI QoL scores remained stable during surveillance. A retrospective exploratory analysis identified SMARC4 and TP53 mutations as independent predictors of aggressive disease [109].

No universally accepted clinical criteria define candidacy for active surveillance (AS) in metastatic ccRCC, and the breadth of eligibility in the prospective cohort reported by Rini et al. illustrates this heterogeneity. Patients often viewed as suboptimal AS candidates—including those with adverse IMDC risk and even brain metastases—were eligible, highlighting the need for more consistent selection tools. We therefore propose pragmatic clinical criteria to identify patients most suitable for AS (Table 3), while recognising that patient preference, values, and capacity for close follow-up are central; some individuals will reasonably choose immediate systemic therapy. Given the high efficacy and quality-of-life gains achievable with contemporary regimens, AS is best suited to patients with minimal or no cancer-related symptoms and no lesions at imminent risk of causing organ-threatening complications. Rapid tumour growth can precipitate oncologic emergencies (e.g., vertebral metastases with cord compression), yet robust models to predict metastatic growth kinetics are lacking [110]. Tumour growth may also be non-linear, often described by Gompertzian dynamics with an early exponential phase followed by deceleration, which warrants particular caution when baseline metastatic burden is substantial [111]. Clinically, lower IMDC scores (0–1) and metachronous relapse ≥ 1 year after local therapy are more frequently associated with indolent behaviour and may favour an AS-first approach. In the Rini et al. trial, median tumour growth during surveillance was 0.09 cm/month (IQR 0.04–0.17) [108]. To operationalise “slow-growing” for practice and research, we use an approximate reference of approximately 0.1 cm/month increase in RECIST 1.1 sum of diameters (SLD), anchored to these prospective data rather than intended as a validated cut-off; where feasible, growth kinetics should be estimated over at least two sequential scans separated by ≥3 months and interpreted in the context of symptoms, lesion location, and overall disease tempo. Because unidimensional RECIST measurements are subject to meaningful inter- and intra-observer variability that can influence apparent growth rates and response categorisation, numeric slopes should not be used in isolation to mandate treatment initiation. Similarly, any lesion-size limits (e.g., “no dominant lesion > 3 cm”) should be stated explicitly as heuristics for low-volume, non–organ-threatening disease rather than standardised criteria.

In the Rini et al. trial, surveillance visits occurred every 3 months in year 1, every 4 months in year 2, and every 6 months thereafter [108]. Clear, validated triggers to end AS are not established. Reasonable criteria for initiating therapy include emergence or worsening of disease-related symptoms, RECIST-consistent progression (particularly rapid or multisite), development of lesions in organ-threatening locations, increase in IMDC risk category, and patient preference. The real-world prospective observational data (MaRCC) demonstrate the feasibility of embedding standardised PRO monitoring during AS, helping to define patient-centred triggers for treatment initiation [107].

**Table 3:** Proposed criteria for active surveillance.

A
Minimal or no symptoms attributable to the tumour (including paraneoplastic syndromes).
No potential complications in the event of rapid tumour growth (e.g., no spinal bone metastases).
Low tumour burden/small metastases (<3 cm of lesion diameter) and indolent growth (≤0.1 cm/month).
Metachronous disease, with time to recurrence ≥ 1 year from previous nephrectomy.
International Metastatic RCC Database Consortium risk score 0–1 points.
Patient preference and adherence.

## 5 Treatment Selection in Favourable-Risk Advanced ccRCC

**Figure 1:**
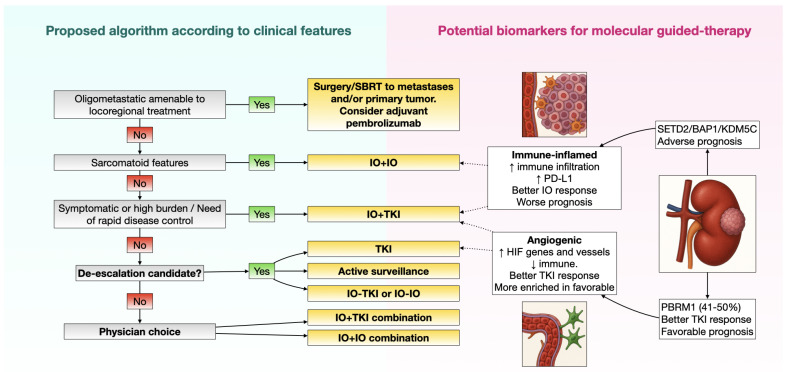
**Proposed treatment-selection framework for IMDC favourable-risk metastatic clear-cell renal cell carcinoma (ccRCC), integrating clinical features and candidate biomarkers. Left panel (clinical algorithm):** Patients with oligometastatic disease amenable to locoregional therapy may be considered for metastasis-directed treatment (surgery and/or stereotactic body radiotherapy [SBRT]) to metastases and/or the primary tumour; if rendered M1-NED after nephrectomy and MDT, adjuvant pembrolizumab may be considered. In patients not suitable for locoregional therapy, sarcomatoid features favour an immune checkpoint inhibitor doublet (IO+IO). Patients who are symptomatic or have high tumour burden requiring rapid disease control are directed toward an IO+TKI regimen. Patients meeting de-escalation criteria (asymptomatic, low-volume, indolent kinetics, and no imminent risk of organ-threatening progression) may be managed with VEGF-TKI monotherapy or active surveillance (AS), with escalation to systemic combination therapy if progression or symptoms develop. When none of the above criteria clearly apply, treatment is individualised based on the physician and patient preference, comorbidities, and access. **Right panel (molecularly guided therapy—investigational):** Two broad transcriptional phenotypes recur across ccRCC classification systems: an immune-inflamed state (higher immune infiltration and PD-L1 expression), associated with poorer baseline prognosis but potentially greater sensitivity to immunotherapy, and an angiogenic state (high HIF/angiogenesis-related gene expression), associated with favourable prognosis and relative VEGF-TKI sensitivity. Selected tumour alterations are shown as candidate prognostic/predictive correlates, including PBRM1 (often linked to more favourable biology) and BAP1/SETD2/KDM5C (often linked to adverse prognosis). These biomarkers are not yet validated to direct routine first-line selection and should be interpreted as hypothesis-generating. *Abbreviations:* AS, active surveillance; IO, immune checkpoint inhibitor; TKI, VEGF receptor tyrosine kinase inhibitor; SBRT, stereotactic body radiotherapy; PD-L1, programmed death-ligand 1; HIF, hypoxia-inducible factor.

First-line therapy for advanced ccRCC comprises VEGF-TKIs and ICIs—anti–PD-1/PD-L1 and anti–CTLA-4 monoclonal antibodies—used alone or in combination. Regimen selection is typically guided by IMDC risk score, comorbidities, and patient preference. Preferred options for intermediate/poor-risk disease include dual ICI (IO+IO; anti–CTLA-4 plus anti–PD-1/PD-L1) or an ICI combined with a VEGF-TKI (IO+TKI; VEGF-TKI plus anti–PD-1/PD-L1). Options for favourable-risk disease include IO+TKI, VEGF-TKI monotherapy, IO+IO, and AS. Guidelines favour IO+TKI [9,10,11] for favourable-risk patients, with TKI monotherapy a less preferred option. Uncritical universal use in all IMDC favourable-risk patients is not evidence based and may constitute overtreatment in carefully selected indolent cases. As the updated CheckMate 214 results showed a non-significant OS trend for nivolumab plus ipilimumab versus sunitinib in this subgroup, IO + IO was included as a preferred regimen in the NCCN guidelines. AS is acknowledged in guidelines, but explicit selection criteria remain undefined. Given the breadth of effective options, management is not one-size-fits-all. Therapy should be individualised, ideally delivered through a multidisciplinary tumour board, with consideration of patient preferences. This work proposes a decision algorithm for favourable-risk disease, including special scenarios (Fig. 1).

**IO+TKI/IO+IO:** The principal advantages of IO+TKI regimens are higher ORRs and a greater probability of complete response (Table 2), which are valuable for symptomatic patients or those requiring rapid tumour control. Cross-trial comparisons between different IO+TKI combinations are inappropriate due to differing populations and designs. TKIs vary in potency, spectrum of kinase inhibition, and toxicity profile; choice should reflect clinician experience and comorbidities [112]. Incorporating checkpoint blockade (IO+IO or IO+TKI) offers the possibility of prolonged disease control and is often preferred in younger, fitter patients with longer life expectancy. The IMDC favourable-risk category likely contains molecular subsets that derive marked benefit from immunotherapy but are not identifiable with current clinical risk models, and later-line IO may be precluded by patient attrition. Disadvantages include higher costs and the risk of immune-related adverse events (irAEs). However, no validated predictive biomarkers reliably identify patients likely to achieve durable benefit, underscoring the limitations of IMDC alone for IO selection in favourable-risk disease.

IO+IO offers deep and durable responses in some patients, a finite treatment course (a fixed 2-year duration is a reasonable strategy, supported indirectly by contemporary IO+TKI trial designs), and avoidance of chronic VEGF-TKI toxicities. These advantages are counterbalanced by the higher rates of immune-related adverse events and lower ORR with a greater proportion of primary PD compared with IO+TKI. Nivolumab plus ipilimumab has a substantially higher immune-mediated toxicity than TKI+IO therapies, with any-grade irAEs in most patients and grade ≥ 3 irAEs in roughly one-third across the RCC series. While IO+TKI regimens exhibit similar or higher overall grade ≥ 3 AE rates (approximately 70–80%) due to the VEGFR–TKI component, grade ≥ 3 immune-mediated events are generally in the 5–10% range [113,114]. Patients with low-volume disease who prioritise durable control and the possibility of treatment-free intervals may be well suited to an IO+IO regimen, acknowledging its lower likelihood of achieving an early response.

Patient comorbidities can help steer regimen selection. Individuals with cardiovascular disease or poorly controlled hypertension, a history or high risk of thromboembolism, clinically significant proteinuria or chronic kidney disease, or likelihood of major surgery are often suboptimal candidates for VEGF-TKIs. Conversely, patients with clinically significant autoimmune disease or a history of solid-organ transplantation face an increased risk of irAEs with checkpoint inhibitors [115]. irAEs are an on-target consequence of immune checkpoint blockade, arising from loss of peripheral tolerance and amplification of autoreactive immunity [116]. This risk–benefit trade-off becomes particularly salient when the absolute efficacy advantage is small, as may occur in selected favourable-risk disease. In IO+TKI regimens, early recognition of toxicity and accurate attribution (immune-mediated versus VEGFR on-target effects) are crucial, as initial management often diverges: irAEs typically require treatment interruption and immunosuppression, whereas TKI toxicities are usually addressed with supportive measures plus dose interruption and/or reduction [117,118].

When irAEs are suspected, infection, disease progression, and alternative aetiologies should be excluded [119,120]. Grade 1 events are often managed symptomatically with close monitoring, while grade ≥ 2 generally warrants holding immunotherapy and initiating corticosteroids, followed by a gradual taper over several weeks to minimise rebound. Endocrinopathies usually require hormone replacement rather than prolonged high-dose steroids, and steroid-refractory toxicity may necessitate organ-specific second-line immunosuppression with multidisciplinary input.

In contrast, VEGFR–TKI adverse effects (e.g., hypertension, diarrhoea, hand–foot syndrome, mucositis, fatigue, proteinuria, transaminitis, hypothyroidism) are best mitigated through proactive monitoring and targeted supportive care, using temporary interruptions and stepwise dose reductions to restore tolerability while preserving efficacy [121,122]. Overall quality of life is broadly similar with IO+TKI and TKI alone, and appears higher with IO+IO than with TKI in the overall population [123].

Sarcomatoid dedifferentiation is present in 5–10% of mRCC cases overall; however, only a small minority of these cases are IMDC favourable risk [124]. In the sarcomatoid subset of the CheckMate-214 trial, nivolumab plus ipilimumab yielded superior outcomes versus sunitinib at 5 years: median OS was 48.6 vs. 14.2 months (HR 0.46), with benefits irrespective of PD-L1 status [125]. IO+TKI combinations are also effective in this phenotype [126].

**TKI monotherapy/AS:** VEGF-TKI alone remains appropriate for patients who do not require maximal response rates (e.g., asymptomatic individuals or those with metachronous, indolent recurrences). Due to extensive clinical experience and well-characterised safety profiles, we favour sunitinib or pazopanib over less established first-line TKIs. Newer agents such as cabozantinib, tivozanib, and axitinib are better reserved for subsequent lines, consistent with their evidence base [127]. AS remains a reasonable alternative for appropriately selected patients with indolent tumour biology (Table 3), particularly where competing comorbidities shorten life expectancy or reduce tolerance for treatment-related toxicity. AS and VEGF-TKI monotherapy are pragmatic approaches for favourable-risk patients in low- to middle-income settings [128,129,130]. Sunitinib and pazopanib present a less expensive alternative to newer TKIs, and immunotherapy can be used in later lines of treatment according to the CheckMate-025 trial [131,132]. Where IO+IO or IO+TKI are accessible, their use should be prioritised for higher-risk disease as absolute benefits are greater.

**Metastatic site:** Traditional risk models do not incorporate metastatic sites, despite clear differences in prognosis [133,134]. Certain metastatic sites, such as pancreatic/glandular regions, lymph nodes only, and lungs, hold better prognoses, making them ideal for de-escalation strategies such as VEGF-TKI and AS. Pancreatic-tropic ccRCC often follows an indolent course and is enriched for angiogenic programs, frequent PBRM1 loss, and relatively fewer aggressive alterations (e.g., BAP1), aligning with VEGF-TKI sensitivity [135]. Hepatic and bone metastases are associated with aggressive biology and worse prognosis [136,137,138]. In such cases, the adverse prognostic impact of liver or bone involvement should override IMDC favourable status; de-escalation strategies such as AS or TKI monotherapy are generally inappropriate.

**Locoregional treatment:** Metastasis-directed therapy (MDT) such as surgery, stereotactic body radiotherapy, and ablation can delay or avoid systemic therapy. Favourable features include ≤ 3–5 lesions amenable to complete ablation/resection, favourable/intermediate IMDC risk, long disease-free interval (>1–2 years), good performance status, and lung-dominant metastases. Patients achieving M1 NED (no evidence of disease) after nephrectomy and MDT have improved OS with adjuvant pembrolizumab following nephrectomy and MDT. AS is also acceptable in late metachronous M1-NED recurrences. Upfront cytoreductive nephrectomy (CN) is no longer routine where systemic therapy is indicated [139], but may be considered in favourable-risk patients. The CARMENA [140,141] and SURTIME trials [142] favoured initial systemic treatment with TKIs. In the current treatment landscape, retrospective series [143] and meta-analyses [144,145] show that CN is consistently associated with better OS in ICI-treated mRCC, but the data are retrospective and subject to confounding factors. Current management usually reserves CN for fit patients with favourable or selected intermediate risk, low metastatic burden, or potentially achievable eradication. Palliative CN remains appropriate for uncontrolled bleeding, pain, or obstruction.

**Treatment duration:** Contemporary trials have largely standardised anti–PD-L1 duration at 2 years; the optimal duration of VEGF-TKI remains undefined. TKIs are traditionally continued until unacceptable toxicity or progression [146]. Data supporting elective ‘drug holidays’ or discontinuation are limited; however, this may be considered in patients with persistent TKI-related toxicity and durable disease control after 2 years of therapy.

## 6 Future Directions

Clinical trials are usually conducted in the general ccRCC population, with favourable-risk patients remaining a minority. This hinders the interpretation of results in this subpopulation, as trials are not sufficiently powered to detect significant differences between groups. The IMDC favourable-risk label identifies a population for whom less intensive strategies (e.g., AS or VEGF-TKI monotherapy) may be appropriate. This label is necessary, but not sufficient, for de-escalation eligibility. Clinical features indicating indolent biology, such as metastatic sites with good prognoses (lungs/lymph nodes/pancreas), slow growth, and minimal symptoms, should guide eligibility in prospective de-escalation studies. Such studies should include standardised escalation triggers, QoL measures, and economic endpoints. Drug exposure reduction strategies are being tested, with new trials exploring alternate dosing of cabozantinib and imaging-guided treatment holidays.

Research in the HIF pathway/angiogenesis genes associated with favourable-risk and ‘angiogenic’ molecular subtypes could lead to actionable targets. A bispecific PD-1/VEGF mAb (ivonescimab/AK112) antibody is under analysis in RCC studies, with a planned Ib/II single-arm trial confined to IMDC favourable-risk, first-line patients (NCT06472895). In the phase III LITESPARK-005 study, belzutifan (an HIF-2α inhibitor which targets the truncal pVHL/HIF axis [29]) improved PFS and ORR (vs. everolimus) in previously treated ccRCC with improved patient-reported outcomes and QoL [147,148]. The favourable toxicity profile and on-target mechanism make HIF-2α inhibitors particularly suitable as future first-line de-escalation strategies in favourable-risk disease. A randomised trial will compare AS versus belzutifan (NCT07023432).

Personalised therapy, in which first-line choice is driven by tumour biology, is under investigation in clinical trials using tumour molecular subgroups. The objective is to avoid unnecessary toxicity and cost by exploiting tractable vulnerabilities. Two prospective programs explicitly test transcriptomic subtyping to guide therapy. The BIONIKK phase II trial assigned treatment according to the French group 35-gene classifier (ccRCC1–4) [58,149]. The ccRCC2 showed ORR 50% with sunitinib versus 51% nivolumab + ipilimumab. The hypothesis-generating data demonstrate the feasibility of first-line personalisation using a transcriptomic subtype and suggest that ccRCC2 is highly TKI-responsive yet may benefit from IO. The ongoing OPTIC RCC phase II trial, a biomarker-guided study, uses RNA-seq clusters derived from IMmotion151 to assign therapy, with angiogenic clusters (1/2) receiving nivolumab + cabozantinib (IO+TKI) [150]. Biologically meaningful ccRCC subtypes can be acted on prospectively, but routine use awaits assay standardisation, solutions to sampling heterogeneity, phase III-level proof of predictive utility, and practical/financial solutions.

Radiopharmaceutical strategies, exemplified by CAIX-targeted ^177^Lu-girentuximab combined with nivolumab+cabozantinib (STARLITE-1), aim to pair tumour-directed radiation with IO+TKI. Cellular therapies are re-entering RCC via CD70-directed platforms (e.g., allogeneic CAR-T ALLO-316) with early signals of activity in heavily pretreated ccRCC [151]. Vaccines are re-emerging with stronger evidence: while a multipeptide vaccine (IMA901) failed to improve survival when added to sunitinib in metastatic ccRCC, personalised neoantigen peptide vaccination in resected high-risk RCC has demonstrated consistent immunogenicity with vaccine-reactive T cells, supporting combination trial designs [152,153]. Next-generation immunomodulators, including engineered IL-2 agonists and PD-L1-VEGF bispecific antibodies, are being developed to intensify antitumor immunity while improving tolerability and potentially overcoming resistance [154,155].

## 7 Conclusions

IO+TKI combinations have reshaped ccRCC care, but most metastatic cases still progress, and effective later-line options remain limited. IMDC risk stratification will continue to guide treatment selection, yet it should not be the sole driver of therapy choice. In favourable-risk disease, the typically indolent course and VEGF sensitivity support TKI monotherapy or AS for carefully selected, asymptomatic patients with low-burden, slow-growing tumours. Given its higher toxicity and cost, IO+TKI should not be a default approach without a consistent overall survival benefit in this subgroup. Prospective trials are needed to test AS, planned treatment breaks, and other de-escalation strategies—particularly in resource-limited settings—while measuring survival, QoL, and cost-effectiveness against continuous therapy. Achieving safe de-escalation will require biomarker-driven personalisation, as clinical prognostic models only indirectly reflect tumour biology; robust predictive biomarkers, especially for immunotherapy benefit; and lower-toxicity novel agents.

## Data Availability

Not applicable.
